# Stanniocalcin-1 Regulates Extracellular ATP-Induced Calcium Waves in Human Epithelial Cancer Cells by Stimulating ATP Release from Bystander Cells

**DOI:** 10.1371/journal.pone.0010237

**Published:** 2010-04-20

**Authors:** Gregory J. Block, Gabriel D. DiMattia, Darwin J. Prockop

**Affiliations:** 1 Institute for Regenerative Medicine at Scott and White Hospital, Texas A&M Health Sciences Center, Temple, Texas, United States of America; 2 London Regional Cancer Program and the Department of Oncology, Biochemistry, University of Western Ontario, London, Ontario, Canada; University of Birmingham, United Kingdom

## Abstract

**Background:**

The epithelial cell response to stress involves the transmission of signals between contiguous cells that can be visualized as a calcium wave. In some cell types, this wave is dependent on the release of extracellular trinucleotides from injured cells. In particular, extracellular ATP has been reported to be critical for the epithelial cell response to stress and has recently been shown to be upregulated in tumors *in vivo*.

**Methodology/Principal Findings:**

Here, we identify stanniocalcin-1 (STC1), a secreted pleiotrophic protein, as a critical mediator of calcium wave propagation in monolayers of pulmonary (A549) and prostate (PC3) epithelial cells. Addition of STC1 enhanced and blocking STC1 decreased the distance traveled by an extracellular ATP-dependent calcium wave. The same effects were observed when calcium was stimulated by the addition of exogenous ATP. We uncover a positive feedback loop in which STC1 promotes the release of ATP from cells *in vitro* and *in vivo*.

**Conclusions/Significance:**

The results indicated that STC1 plays an important role in the early response to mechanical injury by epithelial cells by modulating signaling of extracellular ATP. This is the first report to describe STC1 as a modulator or purinergic receptor signaling.

## Introduction

Stanniocalcin-1 (STC1) is a 247 amino acid protein that is secreted from cells as a glycosylated homodimer. STC1 was originally described as an endocrine regulator of calcium and phosphate homeostasis in fish [1], [2]. In vertebrates, STC1 can regulate mineral metabolism [3] through its modulation of phosphate resorprtion in kidney [4] and the gut [5]. STC1 was also implicated in the uncoupling of oxidative phosphorylation [6], inhibition of macrophage migration *in vitro* [7] and *in vivo* [8], prevention of vascular permeablization [9], and both pro- and anti-apoptotic effects [10], [11], [12]. To date, a mechanism by which STC1 exerts its pleiotropic effects has not been established. More recently, it has become clear that STC1 is a stress responsive factor (Chang et al., 2003), consistent with recent observations that the STC1 transcript is rapidly up-regulated following injurious signals [10], [13], [14].

The role of STC1 in calcium homeostasis and tissue injury suggests it may be involved in the calcium movement and signaling that are triggered by a variety of mechanical and other stresses to cells. For example, the repair of epithelial monolayers following mechanical disruption is dependent on an intercellular calcium wave propagated from the site of disruption to adjacent cells [15], [16], [17], [18], [19]. The propagation of the calcium wave has been attributed either to gap-junction mediated transfer of calcium or low-molecular weight second messengers, or to the release of intracellular nucleotides from the injured cells that then bind to receptors on neighboring cells [20], [21], [22]. Several reports demonstrated that extracellular adenosine triphosphate (ATP) promoted repair in the disrupted cultures by stimulating the migration and proliferation of the epithelial cells [15], [18]. Recently, increased levels of extracellular ATP were visualized in developing tumors *in vivo*, and may contribute to cancer cell survival [23].

Extracellular nucleotides modulate intracellular calcium by binding to a family of receptors called P2 purinergic receptors, each of which has a different affinity for specific nucleotides. There are two sub-classes of P2 receptor: the P2X trimeric gated ion channels and P2Y G-protein coupled receptors (GPCRs). Binding to a P2X receptor leads to a conformational change in the channel that allows the influx of calcium and other ions, whereas P2Y is a G-protein coupled receptor that uses the energy of GTP-hydrolysis to phosphorylate phospolipase C (PLC) following ligand binding. PLC can then cleave the lipid, phosphatidylinositol 4,5-bisphosphate (PIP2), into inositol 1,4,5-trisphosphate (IP3) and diacyl glycerol (DAG). IP3 binds specific calcium ion channels on the endoplasmic reticulum to release calcium into the cytosol. Of the 12 P2Y receptors, only P2Y_2_ has been shown to both bind ATP and couple with the G_q_ G-protein subunit to initiate release of calcium from intracellular stores [24].

Here we demonstrate that pre-treatment of epithelial cells in monolayer culture with STC1 dramatically enhanced calcium wave propagation following mechanical disruption of cultures. The same enhancement was seen when cells were pretreated with STC1 and exposed to exogenous ATP. We provide evidence that STC1 sensitized the cells to ATP upstream of PLC, and that blocking endogenous STC1 using a neutralizing antibody inhibited progression of the calcium wave. This work provides the first evidence that STC1 can modulate an early signaling event following a mechanical injury, and implicates STC1 as a regulator of purinergic receptor signaling.

## Results

### STC1 Enhanced Calcium Wave Propagation Following Mechanical Stimulation of A549 Cells

To investigate the effect of STC1 on injury-induced calcium modulation, confluent monolayers of lung epithelial cells (A549) were mechanically stimulated to initiate a calcium wave. Wave propagation was visualized by pre-incubating the monolayer with a membrane permeable dye that fluoresced upon binding calcium (Fluo-4).

**Figure 1:**
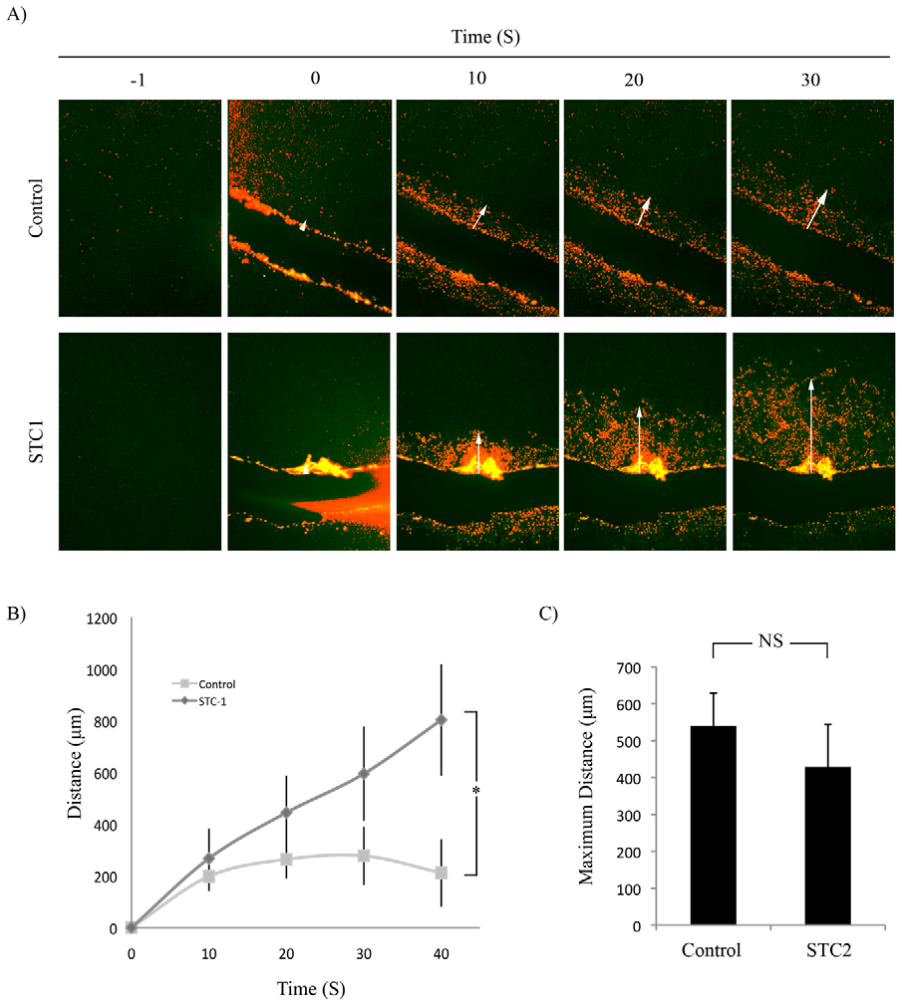
STC1 Enhanced Calcium Wave Propagation Following Mechanical Injury. A) Confluent A549 monolayers were labeled with Fluo-4 and pre-incubated with or without 500 ng/mL STC1 for 10 min prior to mechanical disruption. Shown are images obtained −1, 0, 10, 20 and 30 s post-disruption. Images in each group were manipulated equally using the threshold function in Adobe Photoshop in order to false color the wave for clarity (red). Arrow points to leading edge of wave. B) Mean distance traveled by calcium wave over time in control and STC1 treatment groups from A. Error Bars  =  SD. *  =  p<0.05. n = 5 movies. C) Mean maximum distance reached by calcium wave with or without pretreatment with 500 ng/mL STC2. NS = Not significant. n = 3.

Figure **1**A (Movie S1 and S2) shows representative images of the propagation of a calcium wave originating from the scrape site to adjacent cells over 30 seconds. The distance was quantified by measuring the distance between the cut site and the leading edge of the calcium wave at each time point. Pretreatment of monolayers with STC1 resulted in a 4-fold increase of calcium wave propagation at 40 seconds post-scrape (Figure **1**B) that continued to propagate past the 40 s time point (Movie S2). To test whether the effect was cell type specific, we repeated these studies in a prostate cancer cell line (PC3; Movie S3, S4). STC1 also enhanced calcium wave propagation in the PC3 cells.

We next investigated whether STC2, the only other member of the stanniocalcin family of proteins [25], could affect calcium wave progression under the same conditions. STC2 did not affect the calcium wave (Figure **1**C).

### Calcium Wave Propagation Was Independent of Gap Junctions but Dependent on Extracellular ATP

Calcium wave propagation between adjacent cells has previously been attributed to small molecule transfer by gap junction intercellular communication (GJIC) [20], [26]. To investigate whether GJIC was responsible for calcium wave propagation, A549 monolayers were pretreated with 10, 25, or 50 µM glycyrrhetinic acid (GA), a known inhibitor of connexin-mediated GJIC [27]. The propagation of the calcium wave was not affected, indicating that calcium response was independent of GJIC (Figure S1).

**Figure 2:**
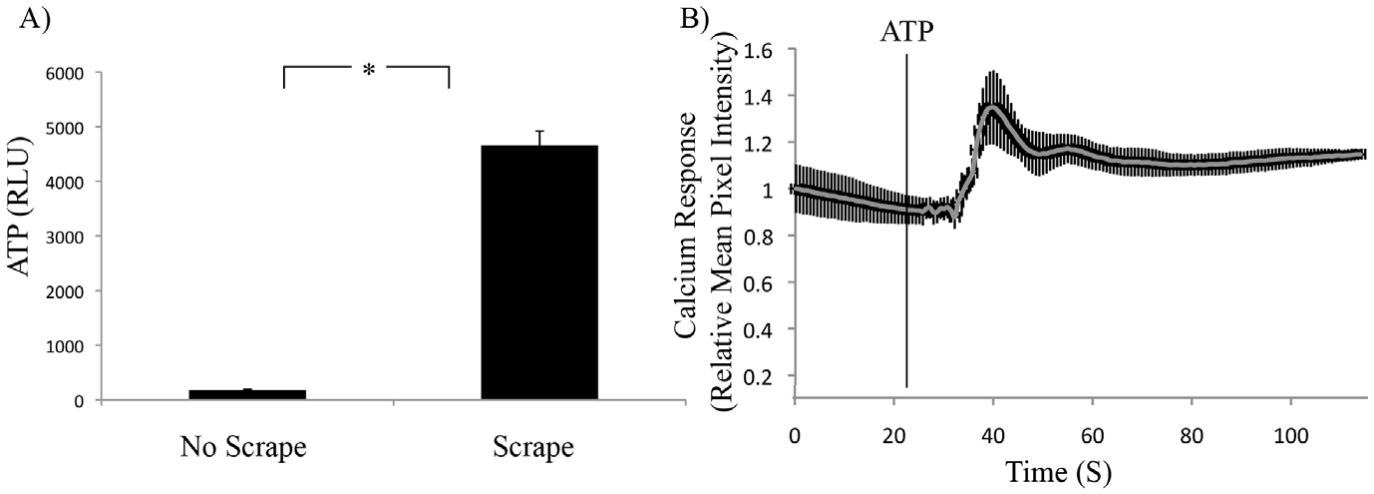
Mechanically Injured Cells Released and Responded to Extracellular ATP. A) Mean ATP content of conditioned media from control or mechanically stimulated A549 monolayers. RLU, relative luciferase units. Error Bars  =  SD. *  =  p<0.05. n = 3. B) ATP (50 µM) alone was added to confluent Fluo-4 labeled A549 cells (vertical line). Calcium response was measured by fluorescent microscopy. n = 4 movies.

**Figure 3:**
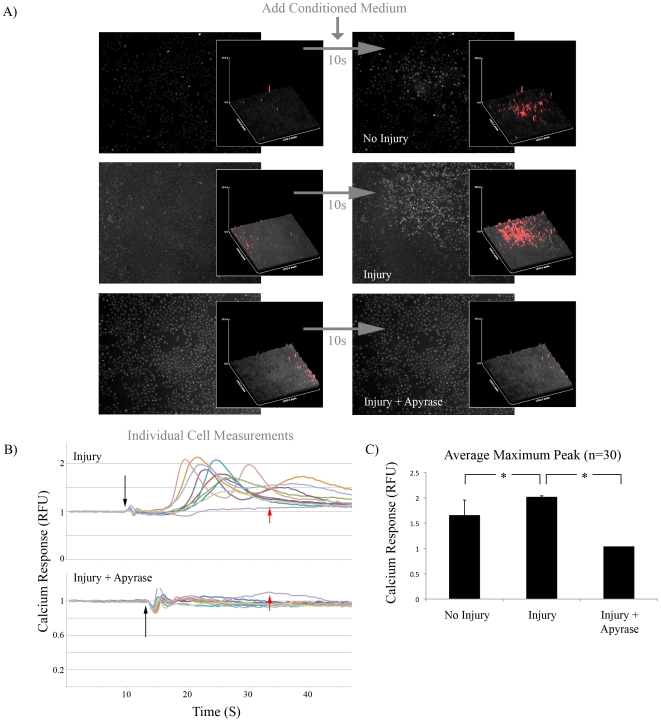
Trinucleotides from Injured Cells Were Required to Initiate Calcium Response. A) Conditioned medium from viable (No Injury), mechanically disrupted (Injury) or mechanically disrupted lysate treated with 250 mU/mL apyrase for 10 min. (Injury + Apyrase) was placed on a confluent layer of Fluo-4 labeled A549 cells. Left column: Before addition of conditioned medium. Right column: 10 s after treatment with conditioned medium. Magnification  = 4×. Inset for each: Pixel intensity profile for entire field of view. Inset images for each treatment group were manipulated equally using the threshold function in Adobe Photoshop in order to false color the peaks for clarity (red). n = 3 movies. B) Calcium response of 10 individual cells following addition of Injury or Injury + Apyrase (250 mU/mL for 10 min) treated conditioned medium. Black Arrow: Addition of conditioned medium. Red Arrow: Background Intensity Measurement. C) Average maximum intensity for individual cells after addition of No Injury, Injury, or Injury + Apyrase conditioned medium. *  =  p<0.05; n = 30.

Others have reported that calcium wave propagation was dependent on the release of ATP from injured cells into the extracellular environment [18], [28]. To confirm that injured cells released ATP, monolayers were scraped with a pipette tip and the conditioned media was measured for ATP content. The conditioned medium contained 400-fold more ATP than conditioned medium from cells that had not been injured (Figure 2A). To confirm that A549 cells were able to respond to ATP by initiating a calcium response, Fluo-4 labeled monolayers were treated with ATP and assayed by live cell fluorescent microscopy. ATP treatment rapidly increased the mean pixel intensity of the measured region of interest (**Figure 2**B). To investigate whether ATP released from scraped cells was responsible for the calcium response in viable cells, media was removed from A549 monolayers and replaced with PBS. The monolayer was left undisturbed to generate ‘No Injury’ conditioned medium, or injured by scraping to generate ‘Injury’ conditioned medium. No Injury and Injury conditioned medium was then placed onto fresh Fluo-4 labeled A549 cells and the calcium response was measured by fluorescent microscopy. Injury conditioned medium induced a more robust calcium response than the No Injury control (Figure 3A; first two rows). Pre-treatment of Injury conditioned medium with an enzyme that hydrolyses trinucleotides to mononucleotides (250 mU/mL apyrase) completely abolished the calcium response. The same data were obtained by measuring the calcium response in individual cells. Again, pre-treatment of Injury conditioned medium with apyrase abolished the calcium response (Figure 3B). Injury conditioned medium also increased the maximum calcium response relative to the No Injury control in individually measured cells, which was abolished by pretreatment with apyrase (Figure 3C).

### STC1 Enhanced ATP-Induced Calcium Response

**Figure 4:**
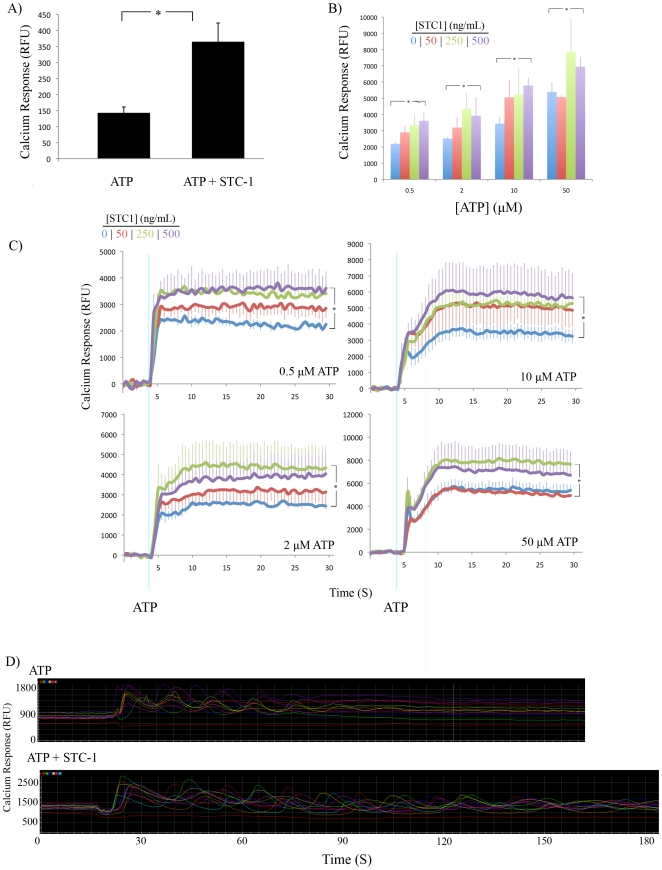
STC1 Enhanced ATP-Induced Calcium Response. A) Mean calcium response of Fluo-4 labeled confluent monolayers of A549 cells were analyzed by live cell microscopy after the addition of 50 µM ATP to control (ATP) or monolayers pretreated with 500 ng/mL STC1 for 10 min (ATP + STC1). Error bars  =  SD. *  =  p<0.05. n = 3 movies. B) Mean calcium response of Fluo-4 labeled A549 monolayers analyzed by fluorescent spectroscopy after addition of various concentrations of ATP and STC1. Data are displayed as average signal intensity at 25 s after addition of ATP. Error bars  =  SD. *  =  p<0.05. n = 4 for each condition. C) Continuous assays from B). n = 3 for B, C. D) Measurement of individual cells from A revealed prolonged calcium oscillations in STC1 pretreated samples.

To investigate whether STC1 affected the ATP-induced calcium response, Fluo-4 labeled A549 monolayers were pretreated with 500 ng/mL STC1 for 10 mins and then stimulated with 50 µM ATP. The calcium response was then measured by fluorescence microscopy. STC1 enhanced the mean fluorescence of a defined region of interest by more than 2-fold (Figure 4A). To corroborate our microscopy results, we quantified the calcium response using fluorescent spectroscopy, which allowed us to test a wider range of concentrations of ATP and STC1. A549 cells were pre-incubated with 0, 50, 250, or 500 ng STC1 for 10 minutes. Following a 4-second baseline reading, ATP was injected automatically into wells at a final concentration of 0.5, 2, 10, and 50 µM. STC1 increased the calcium response in a dose dependent manner; however, at higher doses of ATP, STC1 was required at higher concentrations to have an enhancing effect. Data is displayed as mean endpoint calcium response (Figure 4B) as well as a continuous assay spanning 30 s (Figure 4C).

We noticed that ATP induced calcium oscillations in a small fraction (∼20%) of cells in each condition. When the cells were treated with STC1, these oscillations continued past three minutes, whereas control cells did not oscillate beyond 1.5 minutes. There was no observable difference in the frequency of the oscillations (Figure 4D).

### STC1 Enhanced Calcium Response Upstream of PLC Activation

**Figure 5:**
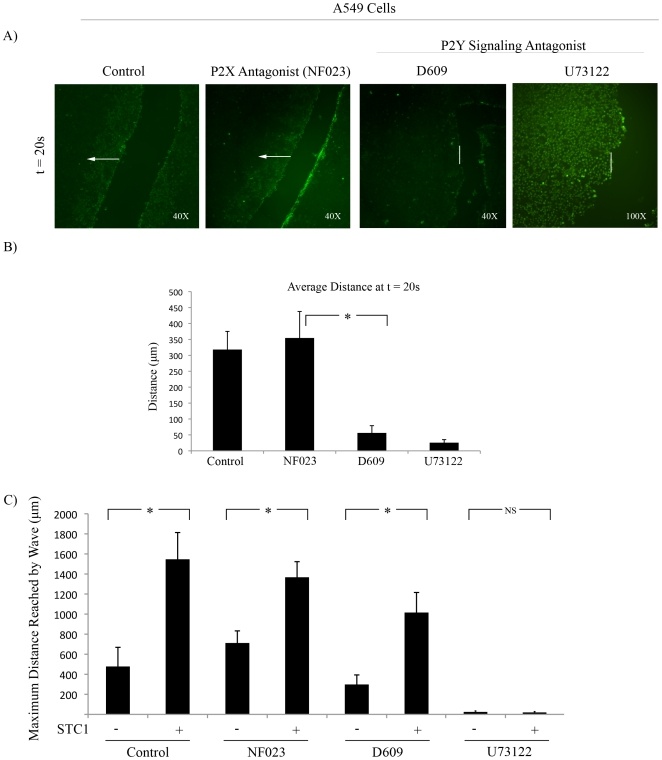
Inhibition of PLC Reduced Mechanical and ATP Induced Calcium Response. A) Fluo-4 labeled A549 cells were mechanically stimulated in the absence (control) or presence of either a P2X inhibitor (50 µM NF023), PI-PLC pathway inhibitor (50 µM D609), or PLC inhibitor (12.5 µM U73122) t = 20 s post-injury. n = 5. B) Quantification of distance traveled by calcium wave from A at t = 20 s. Error bars  =  SD. *  =  p<0.05. n = 5. C) Fluo-4 labeled A549 cells pre-treated for 30 min with NF023, D609, or U73122 were incubated with 500 ng/mL STC1 for 10 min prior to scrape. Maximum distance of the calcium wave is displayed. Error bars  =  SD. *  =  p<0.05. n = 3.

Nucleotide signaling leading to intracellular calcium release can be induced by binding of ATP to either the P2X family of ion channels, or the P2Y G-protein coupled receptors [24]. To investigate which of these pathways were responsible for calcium induction in our model, Fluo-4 labeled A549 monolayers were treated with a P2X antagonist (NF023), or P2Y pathway antagonists (phosphatidylinositol-specific (PI) PLC inhibitor, D609, and PLC inhibitor, U73122). D609 and U73122 reduced the distance and intensity of the calcium wave following mechanical stimulation at 20 s post scrape (Figure 5A,B); however, measurement of maximum distance traveled by the wave revealed that only U73122 completely inhibited the calcium wave (Figure 5C). D609 and U73122 also inhibited the activation of calcium after the addition of exogenous ATP, whereas addition of NF023 had no effect (data not shown). Although D609 reduced the distance and intensity of the calcium wave, pre-incubation of cells with STC1 restored the distance of the wave. STC1 was unable to enhance the calcium wave following incubation with U73122 indicating that STC1 was dependent on canonical PLC activation (Figure 5D).

### STC1 was required for Propagation of Mechanical and ATP-Induced Calcium Wave

**Figure 6:**
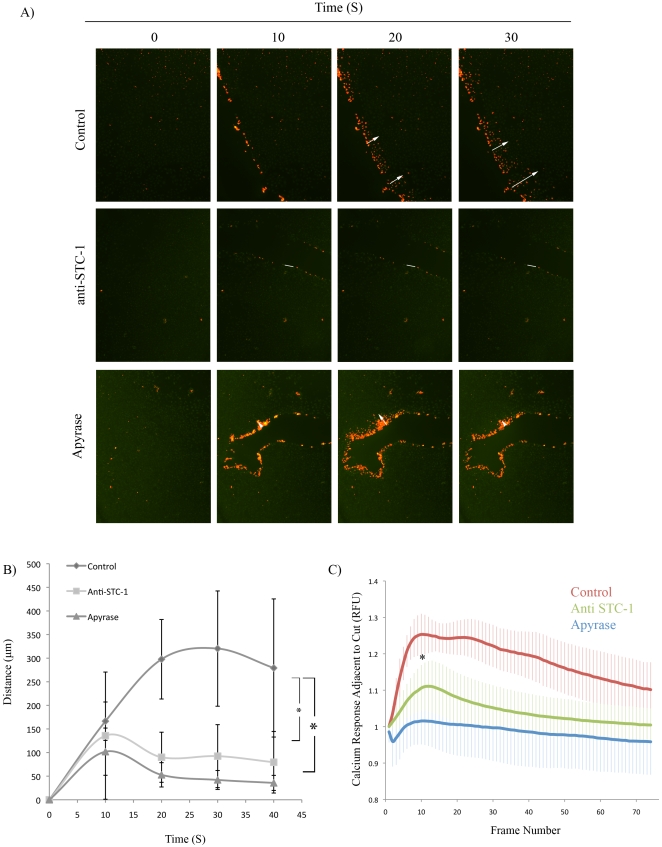
STC1 and Extracellular ATP Were Required for Calcium Wave Propagation following Mechanical Stimulation. A) Fluo-4 labeled A549 monolayers were mechanically stimulated in the presence of isotype antibody (control), an STC1 blocking antibody (anti-STC1; 1 µg/mL) or apyrase (250 mU/mL) and assayed by live cell microscopy. B) Distance of calcium wave propagation from A. Error bars  =  SD. *  =  p<0.05. C) Quantification of signal intensity adjacent to scrape site following mechanical stimulation. Error bars  =  SD. *  =  p<0.05.

The observation that STC1 was acting upstream of PLC prompted us to investigate the effect of endogenous STC1 on calcium wave propagation following mechanical stimulation. A549 cells were pre-incubated with 1 µg/mL polyclonal anti-STC1 antibody that we had previously shown to be effective at blocking STC1 [10]. The cells were then mechanically stimulated and the distance of the calcium wave was measured. Pre-treatment of the cells with anti-STC1 antibody reduced the distance traveled by the calcium wave compared with cells pre-treated with an isotype control antibody. Similarly, pretreatment of the cells with apyrase reduced the distance traveled by the calcium wave (Figure 6A, B). Furthermore, the anti-STC1 treated group displayed a rapid recession of the calcium wave (Figure 6B), as well as decreased calcium response adjacent to the scrape site, as measured by fluorescence microscopy (Figure 6C; Movie S5).

### STC1 Enhanced the Release of ATP from Cells *in vitro* and *in vivo*

**Figure 7:**
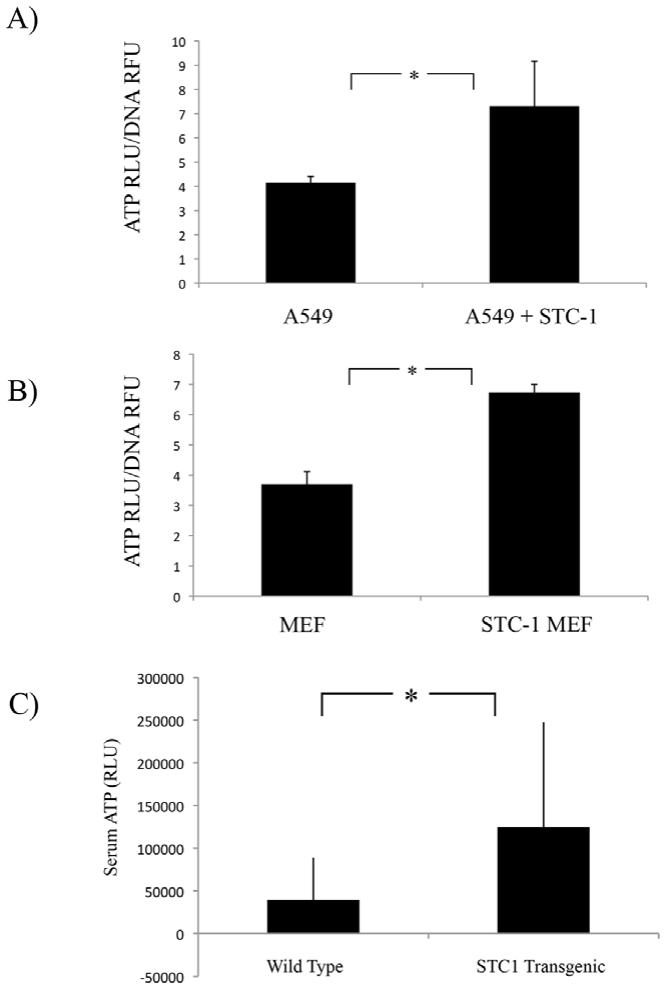
STC1 Enhanced the Release of ATP in vitro and in vivo. A) A549 cells were stimulated with 500 ng/mL STC1 for 10 mins. Conditioned medium was collected and assayed for ATP. Adherent cells were lysed and measured for ATP and DNA content. ATP values were normalized to DNA fluorescence. *  =  p<0.05; n  = 3. B) A549 cells were stimulated with 10 µM ATP for 2, 5 and 10 mins. Assay of conditioned medium and cell lysates of wild type and STC1 over-expressing MEFs. *  =  p<0.05; n  = 3. C) Serum was isolated from wild type and STC1 transgenic mice and assayed for ATP content. *  =  p<0.05; n>17.

We next tested whether STC1 affected the release of ATP from unstimulated epithelial cells. Medium from A549 cells was replaced with serum free medium with or without 500 ng/mL STC1 for 10 mins and an ATP assay was performed. Medium from STC1 treated cells contained 2-fold more ATP (**Figure 7**A). No significant difference was seen in lysates from these cells. ATP levels were normalized to DNA content in each well. Similar results were obtained from mouse embryonic fibroblasts from wild-type and STC1 transgenic mice (**Figure 7**B).

The results suggested the possibility that systemic level of ATP may be elevated in STC1 transgenic mice. To investigate this hypothesis, plasma from wild type and transgenic mice over-expressing STC1 were collected and an ATP assay was performed. Mice expressing the human STC1 transgene showed increased levels of blood ATP compared to wild type controls (**Figure 7**C).

## Discussion

Previous observations suggested that STC1 is stress response protein. The STC1 protein is 80% identical between fish and human, and 98% identical between mice, rats, humans and other mammals [29]. STC1 knockout mice did not display any overt phenotype [30]; however, increased levels of STC1 can alter muscle function and bone development and decrease reproduction [31], [32]. The STC1 mRNA levels were rapidly upregulated during hypoxia and after exposure to various cytokines including tumor necrosis factor alpha, transforming growth factor beta, and fibroblast growth factor-2 (G. Block, unpublished data). Taken together, these observations further support the conclusion that STC1 modulates signaling during the early events of the cellular response to stress.

In order to investigate the role of STC1 in stress, we employed a model of injury in which epithelial monolayers were disrupted with a mechanical stimulus to recapitulate the early events of wound repair [15], [28], [33]. We found that STC1 enhanced the rate and maximum distance of the propagation of an ATP-dependent calcium wave. STC2 did not affect calcium wave progression since pretreatment of cells with STC2 did not alter the maximum distance traveled by the wave. We also deduced that STC1 was dependent on PLC activation in a P2Y G-protein coupled receptor pathway since the PLC inhibitor, U73122, was able to block calcium wave propagation in the presence or absence of STC1. Furthermore, functional blocking of endogenous STC1 using an antibody inhibited the propagation of the calcium wave.

The ubiquity of nucleotide signaling is reflected by the wide distribution of P2 receptors in various cell types [24]. Our observation that STC1 can mediate calcium activation downstream of ATP in lung epithelial cells may also be applicable to other cell types and tissues. For example, the observations that STC1 acted as a selective L-channel inhibitor [34] parallel the early observations that extracellular ATP can slow heart rate after traumatic shock [35], [36]. Furthermore, the involvement of extracellular ATP in regulating macrophage chemotaxis [16], [18], [37], [38], [39] has similarly been defined for STC1 [7]. Moreover, our results suggest that STC1 modulation of purinergic signalling may contribute to the overt phenotypes exhibited by transgenic mice constitutively expressing STC1.

Although STC1 enhances the calcium response downstream of extracellular nucleotides in epithelial cells, the same may not be true in other cell types. For example, STC1 has been shown to activate calcium signaling in endothelial cells [9], but inhibit calcium signaling in response MCP-1 in macrophages [7]. In our assay, STC1 had no effect on calcium dynamics when added alone to A549 cells (Movie S6). Furthermore, ATP may not induce calcium release in all cell types, given that ATP is a potent suppressor of calcium activation in hippocampal neurons [40], [41]. The variable effects of ATP are also reflected in the sensitivity of cells to ATP, as different cell types require different concentrations of extracellular ATP to elicit an effect. Whereas A549 cells respond to as little as 0.5 µM ATP, macrophages generally need >100 µM in order to respond [42]. STC1 may play a role in sensitizing or desensitizing cells to various concentrations of ATP.

The downstream consequences of STC1-mediated activation of an ATP-induced calcium response could have important ramifications for microenvironmental cues leading to normal stress–responses to mechanical and potentially, toxic insults. For example, the role of STC1 in the repair of an epithelial wound may be based on its ability to promote or prevent apoptosis of ATP stimulated cells [43]. The role of STC1 on purinergic signaling from the cell or organelle membranes could have important implications for downstream calcium movement within and between injured cells. This has been challenging in part because of the lack of available bioassays for STC1. The work we have presented here establishes a bioassay for STC1 activity to facilitate a deeper understanding of its structure and function in a variety of cell types and physiological situations.

## Materials and Methods

### Ethics Statement

Mice were housed and used in accordance with protocols approved by the University Council on Animal Care at the University of Western Ontario.

### Cell Culture and Reagents

Human A549 lung cancer epithelial cells and PC3 prostate cancer cells were obtained from the American Tissue Type Culture Collection (Manassas, VA; www.atcc.org). Mouse embryonic fibroblasts and plasma were obtained from wild type and human STC1 transgenic mice as previously described [44]. All cells were maintained in Dulbecco's Minimal Essential Media (DMEM; Invitrogen, Carlsbad, CA; www.invitrogen.com) containing 10% fetal bovine serum (Atlanta Biologicals; Norcross, GA; www.atlantabio.com) and 100 U penicillin/100 µg streptomycin (Invitrogen). Reagents were purchases from the following sources: ATP from Teknova (Hollister, CA; www.teknova.com); apyrase from New England Biolabs (Ipswich, MA; www.neb.com); glycyrrhetinic acid from Sigma-Aldrich (St. Louis, MO; www.sigmaaldrich.com); recombinant human STC1 and STC2 as a FLAG-Tagged fusion protein prepared from human cells from BioVendor (Modrice Czech Republic; www.biovendor.com); goat polyclonal antibodies and mouse monoclonal (clone 380715) anti-STC1 antibody from R&D Systems (Minneapolis, MN; www.rndsystms.com) and the inhibitors NF023 and D609 from Sigma Aldrich.

### Calcium Dye Labeling

To measure changes in intracellular calcium levels, cells were labeled with Fluo-4 No-Wash dye (Invitrogen) according to manufacturer instructions. Briefly, lyophilized dye was reconstituted with 10 mL Assay Buffer (1X calcium and magnesium free HBSS with 20 mM HEPES buffer), and supplemented with 100 µL probenecid acid to prevent the dye from leaking from the cells, according to manufacturer directions. Cells grown in 48 well culture dishes were incubated in 200 µL Fluo-4 for 30 min prior to visualization by live cell microscopy or continuous assay by fluorescence spectroscopy.

### Inhibitor and Antibody Blocking Assays

NF023 (50 µM), D609 (50 µM), U73122 (12.5 µM), and anti-STC1 (1 µg/mL) was added directly to the 200 µL of Fluo-4 assay buffer containing Fluo-4, and incubated alongside the Fluo-4 for 30 min prior to data collection as the cells took up the dye. All inhibitor concentrations were optimized in preliminary experiments using two-fold serial dilutions starting at 100 µM to 50 nM. The effective concentration was determined as the lowest concentration to exert an effect, though in the case of NF023, no effect was ever observed. Initial experiments using glycyrrhetinic acid (5 µM to 100 µM) were performed by pre-incubating the cells for 30 minutes at the same time as they were being incubated with Fluo-4 dye.

### Live Cell Imaging and Mechanical Disruption

Cells were imaged live using a Nikon Ti Eclipse inverted fluorescent microscope (Nikon Instruments inc.; Melville, NY; www.nikoninstruments.com). Cells and microscope were encased in a 37°C environmental chamber (In Vivo Scientific; St Louis, MO; www.invivoscientific.com). Images were taken at 2 frames/sec for at least 1 minute using NIS Elements software. A 4X objective was used (CFI Plan Fluor 4X/.13 17.1 mm; Nikon Instruments). Fluo-4 was excited using a 488 nm excitation filter, and detected at 520 nm emission.

Mechanical stimulation of A549 cells was performed manually by creating a linear scratch with a bent p200 pipette tip (epTIP; Eppendorf, Hamburg Germany, www.eppendorf.com). In order to display the calcium waves in each figure more clearly, all images in each condition were adjusted simultaneously using the Adobe Photoshop threshold function such that pixels above an arbitrarily set threshold turned red.

### Fluorescence Spectroscopy

Fluo-4 labeled cells grown on 48 or 96-well plates were incubated at 37°C in a fluorescence spectrophotometer equipped with two reagent injectors (FluoStar Omega, BMG Labtech, Offenburg Germany; www.bmglabtech.com). The first injector was programmed to deliver increasing doses of ATP after 4 sec baseline reading. The second injector was programmed to deliver reagent buffer prior to the ATP so the volume for each reading remained constant. Wells were assayed at 480 exitation/520 emission. Data from each condition baseline subtracted. Fluorescent intensity measurements were taken every 0.5 sec at 480 activity and analyzed using the BMG Omega Software data analysis software and Microsoft Excel.

### *In Vitro* ATP Assays

ATP was assayed using a luciferase-based protocol according to manufacturer instructions (CellTitre-Glo, Promega; www.promega.com). In order to indirectly quantify cell number, 20 mL of luciferase reagent was supplemented with 50 µL of a DNA intercalating dye (CyQuant; Invitrogen). A single volume of reagent was added to an equal volume of conditioned media. Half of the sample was then assayed for luciferase and the rest for fluorescence (480/520 exitation/emmision) using a plate reader capable of detecting both fluorescence and luminescence (FluoStar Omega, BMG Labtech).

### ATP Assay in Mouse Plasma

Blood was collected from human STC1 transgenic male mice (line 2) [32] and wildtype males (2–4 months of age) of the same genetic background (C57Bl/6 x CBA) using heparin to prevent coagulation. Typically, 300–400 µL was collected per mouse and immediately mixed with stop solution at room temperature to minimize release of ATP from platelets and ATP degradation by ATPase [45]. The stop solution was 3 mM EDTA, 118 mM NaCl, 5 mM KCl, 40 mM tricine buffer, 5 nM nitrobenzyl thioinosine, 10 µM forskolin, 100 µM isobutylmethylxanthine. The blood was immediately centrifuged at 13,000×g for 3 mins at room temperature and 50 µL of plasma was used in duplicate to perform indirect ATP measurements using the CellTiter-Glo Assay (Promega) according to manufacture's instructions. The luminescent signal was measured using a Berthold Lumat LB9507 luminometer and relative light units were converted to ATP concentrations using a standard curve.

### Image and Movie Analysis

Image analysis was performed using Nikon NIS Elements software (Nikon) or ImageJ (rsb.info.nih.gov/ij). In order to quantify the distance of the calcium wave, images were acquired 10, 20, 30 and 40 s post-scrape. For each image, 5 lines were drawn perpendicular to the edge of the scrape to the tip of the calcium wave. Distances of the lines were recorded and averaged for over 5 movies per condition.

### Statistical Analyses

Where two means were being compared, a two-tailed T-test was performed using Microsoft Excel. Under circumstances where more than two means were being compared, null hypothesis was rejected using an analysis of variance. Counts were analyzed using a multi-variable contingency table and chi-squared test using InStat statistical software.

## Supporting Information

Figure S1 Calcium Wave Propagation Was Independent of Gap Junction Intercellular Communication. A549 monolayers were mechanically stimulated following preincubation with with 10, 25, or 50 µM glycyrrhetinic acid and assayed by live cell microscopy. Magnification  = 40×.
(0.30 MB TIF)

Movie S1 Mechanical Stimulation of A549 Epithelial Monolayer Induces Calcium Wave Propagation Images were collected at 2 frames/sec. Playback is 10X original speed. Magnification  = 40×.
(0.21 MB MOV)

Movie S2 STC1 Enhances Calcium Wave Propagation in A549 Cells Images were collected at 2 frames/sec. Playback is 10X original speed. Magnification  = 40×.
(0.20 MB MOV)

Movie S3 Mechanical Stimulation of PC3 Epithelial Monolayer Induces Calcium Wave Propagation. Images were collected at 2 frames/sec. Playback is 10X original speed. Magnification  = 40×.
(0.49 MB MOV)

Movie S4 STC1 Enhances Calcium Wave Propagation in PC3 Cells. Images were collected at 2 frames/sec. Playback is 10X original speed. Magnification  = 40×.
(1.29 MB MOV)

Movie S5 Blocking STC1 with an Antibody Inhibits Calcium Wave Propagation. Images were collected at 2 frames/sec. Playback is 10X original speed. Magnification  = 40×.
(0.12 MB MOV)

Movie S6 STC1 did not affect Calcium Dynamics when added alone to A549 cells. Images were collected at 2 frames/sec. Playback is 10X original speed. Magnification  = 100×.
(0.35 MB MOV)

## References

[1] Wagner GF, Hampong M, Park CM, Copp DH (1986). Purification, characterization, and bioassay of teleocalcin, a glycoprotein from salmon corpuscles of Stannius.. Gen Comp Endocrinol.

[2] Lafeber FP, Herrmann-Erlee MP, Flik G, Wendelaar Bonga SE (1989). Rainbow trout hypocalcin stimulates bone resorption in embryonic mouse calvaria in vitro in a PTH-like fashion.. J Exp Biol.

[3] Olsen HS, Cepeda MA, Zhang QQ, Rosen CA, Vozzolo BL (1996). Human stanniocalcin: a possible hormonal regulator of mineral metabolism.. Proc Natl Acad Sci U S A.

[4] Wagner GF, Vozzolo BL, Jaworski E, Haddad M, Kline RL (1997). Human stanniocalcin inhibits renal phosphate excretion in the rat.. J Bone Miner Res.

[5] Madsen KL, Tavernini MM, Yachimec C, Mendrick DL, Alfonso PJ (1998). Stanniocalcin: a novel protein regulating calcium and phosphate transport across mammalian intestine.. Am J Physiol.

[6] Ellard JP, McCudden CR, Tanega C, James KA, Ratkovic S (2007). The respiratory effects of stanniocalcin-1 (STC-1) on intact mitochondria and cells: STC-1 uncouples oxidative phosphorylation and its actions are modulated by nucleotide triphosphates.. Mol Cell Endocrinol.

[7] Kanellis J, Bick R, Garcia G, Truong L, Tsao CC (2004). Stanniocalcin-1, an inhibitor of macrophage chemotaxis and chemokinesis.. Am J Physiol Renal Physiol.

[8] Huang L, Garcia G, Lou Y, Zhou Q, Truong LD (2009). Anti-Inflammatory and Renal Protective Actions of Stanniocalcin-1 in a Model of Anti-Glomerular Basement Membrane Glomerulonephritis.. Am J Pathol.

[9] Chakraborty A, Brooks H, Zhang P, Smith W, McReynolds MR (2007). Stanniocalcin-1 regulates endothelial gene expression and modulates transendothelial migration of leukocytes.. Am J Physiol Renal Physiol.

[10] Block GJ, Ohkouchi S, Fung F, Frenkel J, Gregory C (2008). Multipotent Stromal Cells (MSCs) are Activated to Reduce Apoptosis in Part by Upregulation and Secretion of Stanniocalcin-1 (STC-1).. Stem Cells.

[11] Wu S, Yoshiko Y, De Luca F (2006). Stanniocalcin 1 acts as a paracrine regulator of growth plate chondrogenesis.. J Biol Chem.

[12] Nguyen A, Chang AC, Reddel RR (2009). Stanniocalcin-1 acts in a negative feedback loop in the prosurvival ERK1/2 signaling pathway during oxidative stress.. Oncogene.

[13] Westberg JA, Serlachius M, Lankila P, Andersson LC (2007). Hypoxic preconditioning induces elevated expression of stanniocalcin-1 in the heart.. Am J Physiol Heart Circ Physiol.

[14] Westberg JA, Serlachius M, Lankila P, Penkowa M, Hidalgo J (2007). Hypoxic preconditioning induces neuroprotective stanniocalcin-1 in brain via IL-6 signaling.. Stroke.

[15] Dignass AU, Becker A, Spiegler S, Goebell H (1998). Adenine nucleotides modulate epithelial wound healing in vitro.. Eur J Clin Invest.

[16] Ehring GR, Szabó IL, Jones MK, Sarfeh IJ, Tarnawski AS (2000). ATP-induced CA2+-signaling enhances rat gastric microvascular endothelial cell migration.. J Physiol Pharmacol.

[17] Wang DJ, Huang NN, Heppel LA (1990). Extracellular ATP shows synergistic enhancement of DNA synthesis when combined with agents that are active in wound healing or as neurotransmitters.. Biochem Biophys Res Commun.

[18] Wesley UV, Bove PF, Hristova M, McCarthy S, van der Vliet A (2007). Airway epithelial cell migration and wound repair by ATP-mediated activation of dual oxidase 1.. J Biol Chem.

[19] Yin J, Xu K, Zhang J, Kumar A, Yu FS (2007). Wound-induced ATP release and EGF receptor activation in epithelial cells.. J Cell Sci.

[20] D'Hondt C, Ponsaerts R, Srinivas SP, Vereecke J, Himpens B (2007). Thrombin inhibits intercellular calcium wave propagation in corneal endothelial cells by modulation of hemichannels and gap junctions.. Invest Ophthalmol Vis Sci.

[21] Homolya L, Steinberg TH, Boucher RC (2000). Cell to cell communication in response to mechanical stress via bilateral release of ATP and UTP in polarized epithelia.. J Cell Biol.

[22] Shabir S, Southgate J (2008). Calcium signalling in wound-responsive normal human urothelial cell monolayers.. Cell Calcium.

[23] Pellegatti P, Raffaghello L, Bianchi G, Piccardi F, Pistoia V (2008). Increased level of extracellular ATP at tumor sites: in vivo imaging with plasma membrane luciferase.. PLoS One.

[24] Erb L, Liao Z, Seye CI, Weisman GA (2006). P2 receptors: intracellular signaling.. Pflugers Arch.

[25] Ishibashi K, Miyamoto K, Taketani Y, Morita K, Takeda E (1998). Molecular cloning of a second human stanniocalcin homologue (STC2).. Biochem Biophys Res Commun.

[26] Boitano S, Sanderson MJ, Dirksen ER (1994). A role for Ca(2+)-conducting ion channels in mechanically-induced signal transduction of airway epithelial cells.. J Cell Sci.

[27] Asl MN, Hosseinzadeh H (2008). Review of pharmacological effects of Glycyrrhiza sp. and its bioactive compounds.. Phytother Res.

[28] Schwiebert EM, Zsembery A (2003). Extracellular ATP as a signaling molecule for epithelial cells.. Biochim Biophys Acta.

[29] Chang AC, Jellinek DA, Reddel RR (2003). Mammalian stanniocalcins and cancer.. Endocr Relat Cancer.

[30] Chang AC, Cha J, Koentgen F, Reddel RR (2005). The murine stanniocalcin 1 gene is not essential for growth and development.. Mol Cell Biol.

[31] Filvaroff EH, Guillet S, Zlot C, Bao M, Ingle G (2002). Stanniocalcin 1 alters muscle and bone structure and function in transgenic mice.. Endocrinology.

[32] Varghese R, Gagliardi AD, Bialek PE, Yee SP, Wagner GF (2002). Overexpression of human stanniocalcin affects growth and reproduction in transgenic mice.. Endocrinology.

[33] Khakh BS, North RA (2006). P2X receptors as cell-surface ATP sensors in health and disease.. Nature.

[34] Sheikh-Hamad D, Bick R, Wu GY, Christensen BM, Razeghi P (2003). Stanniocalcin-1 is a naturally occurring L-channel inhibitor in cardiomyocytes: relevance to human heart failure.. Am J Physiol Heart Circ Physiol.

[35] Drury AN, Szent-Gyorgyi A (1929). The physiological activity of adenine compounds with especial reference to their action upon the mammalian heart.. J Physiol.

[36] Kalckar HM, Lowry OH (1947). The Relationship Between Traumatic Shock and the Release of Adenylic Acid Compouns.. Am J Physiol.

[37] Satterwhite CM, Farrelly AM, Bradley ME (1999). Chemotactic, mitogenic, and angiogenic actions of UTP on vascular endothelial cells.. Am J Physiol.

[38] Janssens R, Boeynaems JM (2001). Effects of extracellular nucleotides and nucleosides on prostate carcinoma cells.. Br J Pharmacol.

[39] Klepeis VE, Weinger I, Kaczmarek E, Trinkaus-Randall V (2004). P2Y receptors play a critical role in epithelial cell communication and migration.. J Cell Biochem.

[40] Kawamura M, Gachet C, Inoue K, Kato F (2004). Direct excitation of inhibitory interneurons by extracellular ATP mediated by P2Y1 receptors in the hippocampal slice.. J Neurosci.

[41] Koizumi S, Fujishita K, Tsuda M, Shigemoto-Mogami Y, Inoue K (2003). Dynamic inhibition of excitatory synaptic transmission by astrocyte-derived ATP in hippocampal cultures.. Proc Natl Acad Sci U S A.

[42] Gordon JL (1986). Extracellular ATP: effects, sources and fate.. Biochem J.

[43] Weinger I, Klepeis VE, Trinkaus-Randall V (2005). Tri-nucleotide receptors play a critical role in epithelial cell wound repair.. Purinergic Signal.

[44] Gagliardi AD, Kuo EY, Raulic S, Wagner GF, DiMattia GE (2005). Human stanniocalcin-2 exhibits potent growth-suppressive properties in transgenic mice independently of growth hormone and IGFs.. Am J Physiol Endocrinol Metab.

[45] Gorman MW, Marble DR, Ogimoto K, Feigl EO (2003). Measurement of adenine nucleotides in plasma.. Luminescence.

