# Supplementary figures and images for: Stanniocalcin-1 Regulates Extracellular ATP-Induced Calcium Waves in Human Epithelial Cancer Cells by Stimulating ATP Release from Bystander Cells
Source: PLoS One. 2010 Apr 20;5(4):e10237. doi: 10.1371/journal.pone.0010237 (PMC2857883; doi:10.1371/journal.pone.0010237)

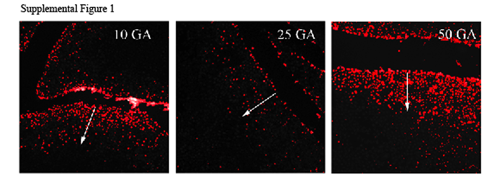

Supplement: Figure S1 — Calcium Wave Propagation Was Independent of Gap Junction Intercellular Communication. A549 monolayers were mechanically stimulated following preincubation with with 10, 25, or 50 µM glycyrrhetinic acid and assayed by live cell microscopy. Magnification = 40×. (0.30 MB TIF) [file pone.0010237.s001.tif]
